# Seroepidemiology of Human Tularemia—Systematic Review and Meta-analysis of Seroprevalence Studies

**DOI:** 10.1093/ofid/ofad636

**Published:** 2023-12-14

**Authors:** Chantal Mattatia, Philipp K A Agyeman, Nina Schöbi, Simon Aebi, Andrea Duppenthaler, Michael Büttcher, Christoph Aebi

**Affiliations:** Division of Pediatric Infectious Disease, Department of Pediatrics, Bern University Hospital, University of Bern, Bern, Switzerland; Division of Pediatric Infectious Disease, Department of Pediatrics, Bern University Hospital, University of Bern, Bern, Switzerland; Division of Pediatric Infectious Disease, Department of Pediatrics, Bern University Hospital, University of Bern, Bern, Switzerland; Division of Pediatric Infectious Disease, Department of Pediatrics, Bern University Hospital, University of Bern, Bern, Switzerland; Risk and Resilience Team, Center for Security Studies (CSS), Eidgenössische Technische Hochschule (ETH), Zurich, Switzerland; Division of Pediatric Infectious Disease, Department of Pediatrics, Bern University Hospital, University of Bern, Bern, Switzerland; Paediatric Infectious Diseases Unit, Department of Paediatrics, Children's Hospital Lucerne, Lucerne Cantonal Hospital, Lucerne, Switzerland; Faculty of Medicine and Health Sciences, University Lucerne, Lucerne, Switzerland; Paediatric Pharmacology and Pharmacometrics Research Center, University Children's Hospital Basel, Basel, Switzerland; Division of Pediatric Infectious Disease, Department of Pediatrics, Bern University Hospital, University of Bern, Bern, Switzerland

**Keywords:** seroepidemiology, seroprevalence, subclinical, systematic review, tularemia

## Abstract

**Background:**

Seroepidemiologic studies of human tularemia have been conducted throughout the northern hemisphere. The purposes of this study were (1) to provide an overview of *Francisella tularensis* seroprevalence data, and (2) to generate an estimate of the proportion of study participants whose infection remained subclinical.

**Methods:**

We conducted a systematic review of *F tularensis* seroprevalence studies according to the PRISMA (Preferred Reporting Items for Systematic Reviews and Meta-Analyses) guidelines. We searched PubMed, Embase, and Web of Science covering the period from 1951 to 2023.

**Results:**

The weighted pooled seroprevalence among 44 486 participants recruited in 52 studies was 3.7% (95% confidence interval [CI], 2.7–5.1). Reported seroprevalences ranged between 0.2% and 31.3%. Occupational activities associated with an increased likelihood of exposure (risk ratio, 3.51 [95% CI, 3.2–3.86]) and studies from North America versus Europe and Asia (4.53 [4.15–4.94]) were associated with significantly increased seropositive rates. Twenty-eight data sets (47%) reported clinical information on a total of 965 seropositive participants. The weighted pooled estimate for subclinical seropositivity was 84.4% (95% CI, 72.9%–991.7%). Studies from *F tularensis* type A areas (risk ratio, 0.37 [95% CI, .27–.51) and studies from sites where pulmonary tularemia prevailed (0.38 [.28–.51]) reported lower subclinical seropositivity rates than studies from type B areas and from areas of predominance of (ulcero)glandular or oropharyngeal tularemia, respectively.

**Conclusions:**

Throughout the northern hemisphere, only a small proportion of study participants showed serologic evidence of exposure to *F tularensis.* Eight of 10 seropositive participants had no historical evidence of past clinical tularemia.

## Teaser

The weighted seroprevalence of human antibodies against *Francisella tularensis* in endemic areas of the northern hemisphere was 3.7%. Eight of 10 seropositive participants had no historical evidence of past clinical tularemia.

Human tularemia is a bacterial zoonosis caused by *Francisella tularensis*, a small, gram-negative coccobacillus with the capacity to infect a wide range of mammals, arachnids, insects and other animals. There are 2 main subspecies. Type A (*F tularensis* subsp *tularensis*) is mainly restricted to North America, although a few strains have been isolated in Europe [1]. Type B (*F tularensis* subsp *holarctica*) is distributed throughout the Northern hemisphere and has also been isolated in Australia [2]. Modes of acquisition in humans are diverse and include arthropod bites (ticks, mosquitos), ingestion of contaminated freshwater or soil, direct contact with infected live or dead animals, and inhalation of contaminated aerosols. Accordingly, clinical manifestations vary and include (ulcero)glandular, oropharyngeal, typhoidal, and pulmonary manifestations [3].

While both the molecular pathogenesis of tularemia [4] and the clinical manifestations in humans have been studied in detail [3, 5], important questions remain unanswered. An issue of particular interest to clinicians is the likelihood of subclinical infection among exposed individuals (ie, asymptomatic or oligosymptomatic, medically unattended infection). Some authors have postulated that the majority of infections remain undetected [6], while others believe that most cases cause a distinct clinical syndrome [7, 8]. A comprehensive review of the available data is lacking. Such information is important, however, as it may elucidate whether subclinical infection as opposed to clinically overt disease is the typical human response to *F tularensis* exposure. It may assist in the clinical interpretation of diagnostic test results and contribute to what is known on how effectively the human immune system deals with *F tularensis*.

One means of addressing this question is to examine seroprevalence studies. Seropositive individuals without a history of clinical disease compatible with tularemia can be considered to have experienced subclinical infection. If they represent the majority of seropositive individuals, it follows that clinical disease cannot usually be explained by pathogen virulence alone but requires a particular set of additional conditions for it to occur. This may be particularly relevant in geographic areas, where the less virulent type B circulates [9]. Clinical disease could then be considered as evidence of some sort of immune compromise around the time of infection. Evidently, alternative explanations are possible and include the mode of acquisition, the infectious dose, strain-specific virulence determinants, and genetic predisposition, which may affect the extent of clinical disease.

Thus, the purposes of this study were (1) to generate an overview of *F tularensis* seroprevalence rates reported from endemic areas worldwide and (2) to generate an estimate of the proportion of human tularemia cases identified by detectable serum antibodies that had no history of past clinical manifestations suggestive of or confirmed to be clinical tularemia (subclinical cases). Because each study usually identifies only a handful of seropositive individuals, we conducted a systematic review of tularemia seroprevalence studies between the 1940s and 2023, in accordance with the PRISMA (Preferred Reporting Items for Systematic Reviews and Meta-Analyses) guidelines for systematic reviews [10].

## METHODS

### Data Source and Search Strategy

PubMed (1946 to present), Embase (1947 to present) and Web of Science (1921 to present) were searched using the following search term combinations: (Tularemia OR *Francisella*) AND (seroprevalence OR seroepidemiolog*); (Tularemia OR *Francisella*) AND antibody AND prevalence; antibod* prevalence tulare* human. Refinements were added as needed. The list of references of each retrieved article was searched for additional suitable articles. No language limitations applied. Studies written in languages other than English, German, or French were full text translated using the web-based translator DeepL® Pro (www.deepl.com). The generated list of articles was screened by title and abstract independently by 2 authors (C. M. and C. A.), who applied the predefined inclusion/exclusion criteria (see below). Discrepancies were resolved by consensus.

### Inclusion and Exclusion Criteria

We included studies, research letters, and abstracts that reported original data on the prevalence of serum antibodies against *F tularensis* in humans. No restriction applied regarding the population studied (eg, geographic location, general vs risk populations, age, sex, ethnicity) and outbreak versus nonoutbreak time periods of serum sampling. Publications were included if published before 30 April 2023. We excluded articles without original data or duplicating previously published data, those focusing on clinical cases, and those without methodologic description of antibody detection tests used. Studies reporting a seroprevalence of 0 were recorded but excluded from analyses.

### Quality Assessment

We next assessed all retrieved studies using a critical appraisal checklist adapted from the Joanna Briggs Institute checklist for prevalence studies [11, 12] (Supplementary DataFile 1 and Supplementary Table 1). We included studies that scored ≥5 of 7 points (>70%) in the 7-question checklist. In addition, we devised a list of criteria identifying high-specificity serologic testing for use in subanalyses (Supplementary Data File 1 and Supplementary Table 2).

### Data Retrieved From Selected Articles

The following data were extracted from all articles included in the analysis: study year and location, population characteristics (age, sex, and risk factors for *F tularensis* exposure), use of a study questionnaire for participants, serologic tests used, tests for cross-reactivity used, cutoff values defining seropositivity, number and proportion of participants testing positive, number and proportion of “subclinical” participants testing positive, and narrative clinical description of participants testing positive.

All patient data used in this systematic review were previously published. According to the local ethics committee, informed consent is not required for systematic reviews.

### Definition of Subclinical Infection

For the purpose of this study, we defined a subclinical participant as an individual who was seropositive for *F tularensis* and explicitly asymptomatic at the time of serum sampling and had a medical history lacking episodes of known tularemia or tularemialike illness.

### Statistical Analysis and Software Used

Pooled counts and weighted proportions of participants seropositive for *F tularensis* were calculated for the entire set of studies and subgroups of interest. Weighted proportions were calculated using both a fixed-effects model and a random-effects model using restricted maximum likelihood estimation of the between-study variance [13], because of the expected occurrence of substantial heterogeneity between the studies (eg, geographic region, risk of exposure to *F tularensis*), other than sampling errors. No limit for heterogeneity as expressed by the *I*² statistic applied. Forest plots are presented with a 95% confidence interval (CI) and group size. Weighted seroprevalences rates for subgroup analyses and risk ratios (RRs) were calculated using the same model when the comparative data sets were complete. When incomplete, the RRs were calculated from the weighted seroprevalences directly. For linear correlation analysis, the Pearson correlation coefficient was calculated. Funnel plots were constructed by plotting the log odds against the study size. As recommended by Hunter et al [14] for meta-analyses of proportion studies (Supplementary Figure 3). The R software package (version 4.0.3; https://www.R-project.org/) and VassarStats software (www.vassarstats.net) were used for analysis. The map (Supplementary Figure 2) was created using ArcGIS, which is intellectual property of Esri (www.esri.com).

## RESULTS

### Bibliographic Data and Study Settings

**Figure 1.**
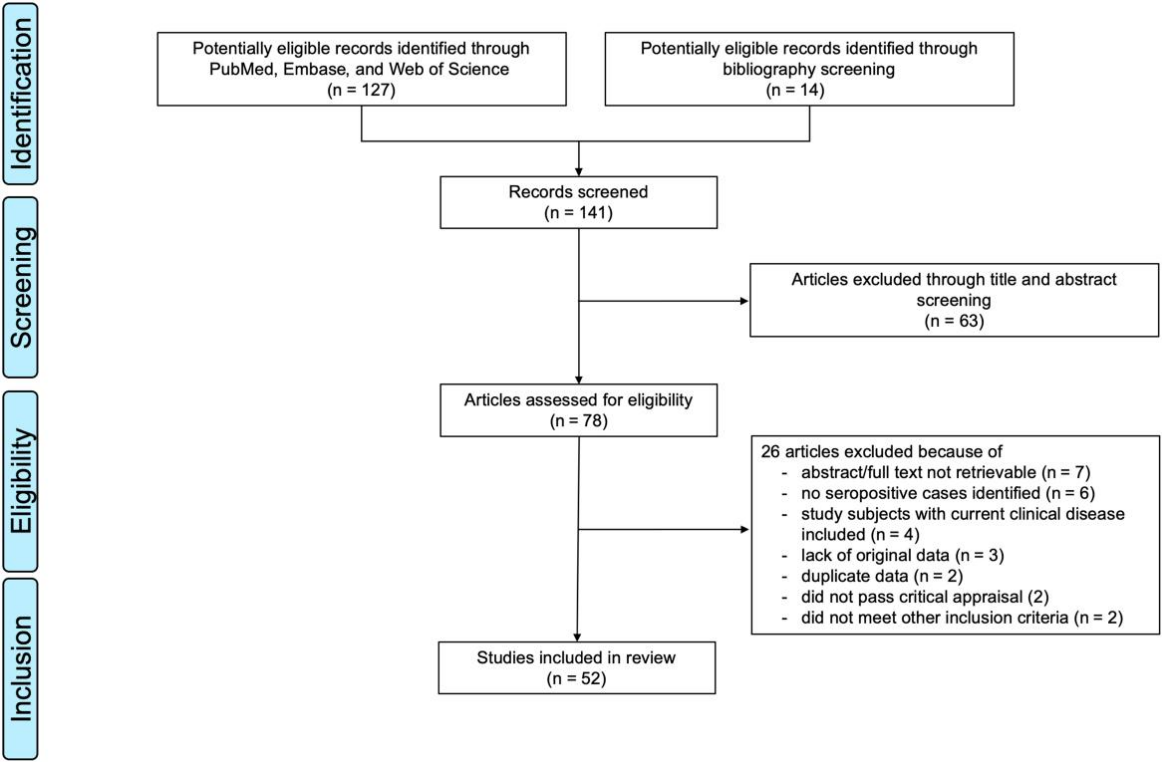
Bibliographic search and selection flow diagram adapted from the PRISMA (Preferred Reporting Items for Systematic Reviews and Meta-Analyses) statement [22].

We identified 52 articles fulfilling the predefined selection criteria. A flowchart detailing the selection process adapted from PRISMA [15] is provided in Figure 1. Six additional seroprevalence studies extracted during the search process were excluded, because no seropositive individuals were identified [16–21]. Seven selected articles provided 2 data sets [6, 23–28], resulting in a total of 59 data sets (Tables 1 and 2). The distribution of the publication years over time is shown in Supplementary Figure 1 (Supplementary Data File 1). The geographic distribution of study countries is displayed in Supplementary Figure 2 (Supplementary Data File 1). Key study settings are summarized in Table 1. Details of each study are provided in Supplementary Data File 2.

**Table 1.** Characteristics of Human *Francisella tularensis* Seroprevalence Studies Included in Review

Characteristic	Studies, No. (%) (N = 52)^a^
Studies with 2 data sets	7 (13)
No. of data sets	59
Geography	
Study site (world region)	
North America	13 (21)
Eastern Europe	4 (7)
Northern Europe	4 (7)
Western Europe	11 (21)
Middle East	18 (34)
East Asia	2 (4)
Site in *F tularensis* type B region	50 (96)
Site in *F tularensis* type A region	2 (4)
Participant characteristics	
Age span specified	52 (100)
Participants <15 y of age included	10 (19)
All participants <18 y of age	1 (2)
Sex distribution of seropositive individuals specified	28 (54)
Risk factors for *F tularensis* exposure identified	33 (64)
Clinical information on seropositive participants provided	28 (56)
Participant questionnaire used	39 (75)
Serology for *F tularensis*	
Primary antibody test used	
TAT	16 (36)
MAT	12 (23)
ELISA	19 (36)
Other	5 (8)
Confirmatory secondary test(s) used	12 (23)
Cross-reactivity against *Brucella* sp tested	26 (50)
High-specificity test strategy used	39 (75)
Non–high-specificity test strategy used	13 (25)

Abbreviations: ELISA, enzyme-linked immunosorbent assay; *F tularensis, Francisella tularensis*; MAT, microagglutination test; TAT, tube agglutination test.

^a^Data represent no. (%) of studies unless otherwise specified.

**Table 2.** Human *Francisella tularensis* Seroprevalence Studies Included in Systematic Review

Author(s) (Year)		All Participants			Seropositive Participants				Antibody Test(s)
	Country	Ages, y	No.	Male/Female, No.	No.	%	Clinically Evaluated, No.	Subclinical, No.	
Wood (1951) [29]	Canada	2–88	2942	1623/1319	344	11.7	344	344	TAT^a^
Greenberg and Blake (1957) [30]	Canada	3–93	797	…	58	7.3	…	…	TAT^a^
Greenberg and Blake (1958) [31]	Canada	0–78	1031	…	139	13.5	…	…	TAT^a^
Philip et al (1962) [32]	USA	All ages	115	…	33	28.7	…	…	TAT
	USA	18–65	793	793/0	60	7.6	20	15	TAT
Philip et al (1967) [23]	USA	15–84	344	332/12	45	13.1	45	42	TAT
Dahlstrand et al (1971) [8]	Sweden	All	1201	…	71	5.9	71	20	TAT^a^
Haug and Pearson (1972) [24]	Norway	13–17	815	…	11	1.3	…	…	TAT (Widal)
	Norway	Adults	55	…	3	5.5	3	0	TAT (Widal)
Koskela and Herva (1982) [33]	Finland	Adults	1072	…	7	0.7	7	6	TAT^a^
Liles and Burger (1993) [34]	USA	Adults	14	…	2	14.3	…	…	TAT^a^
Lévesque et al (1995) [25]	Canada	40 ± 12	165	157/8	4	2.4	4	4	LAT
	Canada	Adults	165	…	1	0.6	…	…	LAT
Aquilini et al (2000) [35]	Italy	21–65	507	507/0	13	2.6	13	13	IF
Feldman et al (2003) [6]	USA	Adults	132	104/28	12	9.1	12	8	MAT
	USA	Adults	310	154/156	1	0.3	…	…	MAT
Gutiérrez et al (2003) [36]	Spain	14–92	4825	2324/2486	9	0.2	…	…	MAT
Deutz et al (2003) [37]	Austria	Adults	149	146/3	5	3.4	…	…	MAT
Porsch-Ozcürümez et al (2004) [38]	Germany	18–79	6632	…	15	0.2	…	…	ELISA + WB
Schmitt et al (2005) [39]	Germany	Adults	1149	…	4	0.3	…	…	ELISA + WB
Gürcan et al (2006) [40]	Turkey	All	266	…	10	3.8	10	3	MAT
Dedeoglu Kilinc et al (2007) [41]	Turkey	6–92	1782	1213/569	5	0.3	…	…	MAT
Campagna et al (2011) [42]	Canada	>15	249	105/146	42	16.9	42	33	TAT
Lévesque et al (2007) [43]	Canada	Adult	50	22/28	2	4.0	2	1	TAT
Jenzora et al (2008) [44]	Germany	Adults	286	…	5	1.7	5	3	ELISA + WB + IFA
Splettstoesser et al (2009) [45]	Germany	10–65	2416	1169/1263	56	2.3	…	…	ELISA + WB
Bazovska et al (2010) [46]	Slovakia	Adults	299		11	3.7	11	11	TAT^a^
Wölfel et al (2010) [47]	Mongolia	Adults	765	670/95	13	1.7	…	…	ELISA and/or IF
Tatman Otkun et al (2011) [48]	Turkey	0.5–76	115	60/55	36	31.3	36	34	TAT
Yazgi et al (2011) [49]	Turkey	16–77	240	134/106	5	2.1	5	4	ELISA + MAT
Sampasa-Kanyinga et al (2012) [50]	Canada	>18	264	110/157	48	18.2	48	39	TAT
Messier et al (2012) [51]	Canada	18–74	917	…	173	18.9	…	…	TAT
Yesilyurt et al (2012) [52]	Turkey	18–67	64	64/0	4	6.3	4	2	MAT + ELISA
Clark et al (2012) [53]	Azerbaijan	>18	796	347/449	123	15.5	123	122	ELISA
Tobudic et al (2014) [54]	Austria	18–60	546	534/12	3	0.5	…	…	ELISA
Esmaeili et al (2014) [55]	Iran	>18	184	184/0	12	6.5	12	…	ELISA
Esmaeili et al (2014) [56]	Iran	>18	250	206/44	36	14.4	…	…	ELISA
Khoshdel et al (2014) [57]	Iran	2–18	183	89/94	11	6.0	11	11	ELISA
Zukiewicz-Sobczak et al (2014) [58]	Poland	35–55	216	176/40	7	3.2	0	…	ELISA
Bayram et al (2015) [59]	Turkey	18–93	495	152/343	18	3.6	18	15	MAT
Zákutná et al (2015) [60]	Slovakia	Adults	124	77/47	5	4.0	…	…	ELPAGA + WB
Rossow et al (2015) [7]	Finland	30–92	1045	481/564	16	1.5	16	15	ELISA + WB
Jurke et al (2015) [61]	Germany	18–66	722	569/153	29	4.0	…	…	ELISA + WB
Gazi et al (2016) [62]	Turkey	49 ± 17	324	156/168	23	7.1	23	23	ELISA
Büyük et al (2016) [63]	Turkey	>15	201	178/23	15	7.5	15	15	MAT + ELISA
Rigaud et al (2016) [64]	France	17–81	2875	2916/59	164	5.7	…	…	TAT + ELISA
De Keukeleire et al (2017) [26]	Belgium	25–72	148	128/20	3	2.0	…	…	ELISA + ICT
	Belgium	18–68	402	118/90	2	0.5	…	…	ELISA + ICT
Akhvlediani et al (2018) [27]	Georgia	18–59	500	476/13	10	2.0	…	…	MAT
	Georgia	18–65	697	310/387	35	5.0	…	…	MAT
Esmaeiliet al (2019) [28]	Iran	30–50	144	144/0	4	2.8	…	…	ELISA
	Iran	27–53	145	145/0	7	4.8	…	…	ELISA
Esmaeiliet al (2019) [65]	Iran	18–78	360	275/85	10	2.8	10	10	ELISA
Harrist et al (2019) [66]	USA	…	23	13/10	3	13.0	3	2	MAT
Takeda et al (2019) [67]	Japan	18–90	1152	…	12	1.0	12	12	RSA
Özdemir et al (2019) [68]	Turkey	20–80	360	180/180	27	7.5	…	…	ELISA
Obaidat et al (2020) [69]	Jordan	All	828	339/489	64	7.7	…	…	ELISA
Karatas Yeni et al (2022) [70]	Cyprus	>18	430	…	4	0.9	…	…	MAT
Davarci et al (2023) [71]	Turkey	2–89	410	226/184	6	1.5	…	…	MAT

Abbreviations: ELISA, enzyme-linked immunosorbent assay; ELPAGA, enzyme linked protein A/G assay; ICT, immunochromatography test; IF, immunofluorescence assay; LAT, latex agglutination test; MAT, microagglutination test; RSA, rapid slide agglutination; TAT, tube agglutination test; WB, Western blot.

Blank cells indicate that no data are available.

^a^The term “agglutination reaction” is used in the original publication.

### Pooled Seroprevalence Data

**Figure 2.**
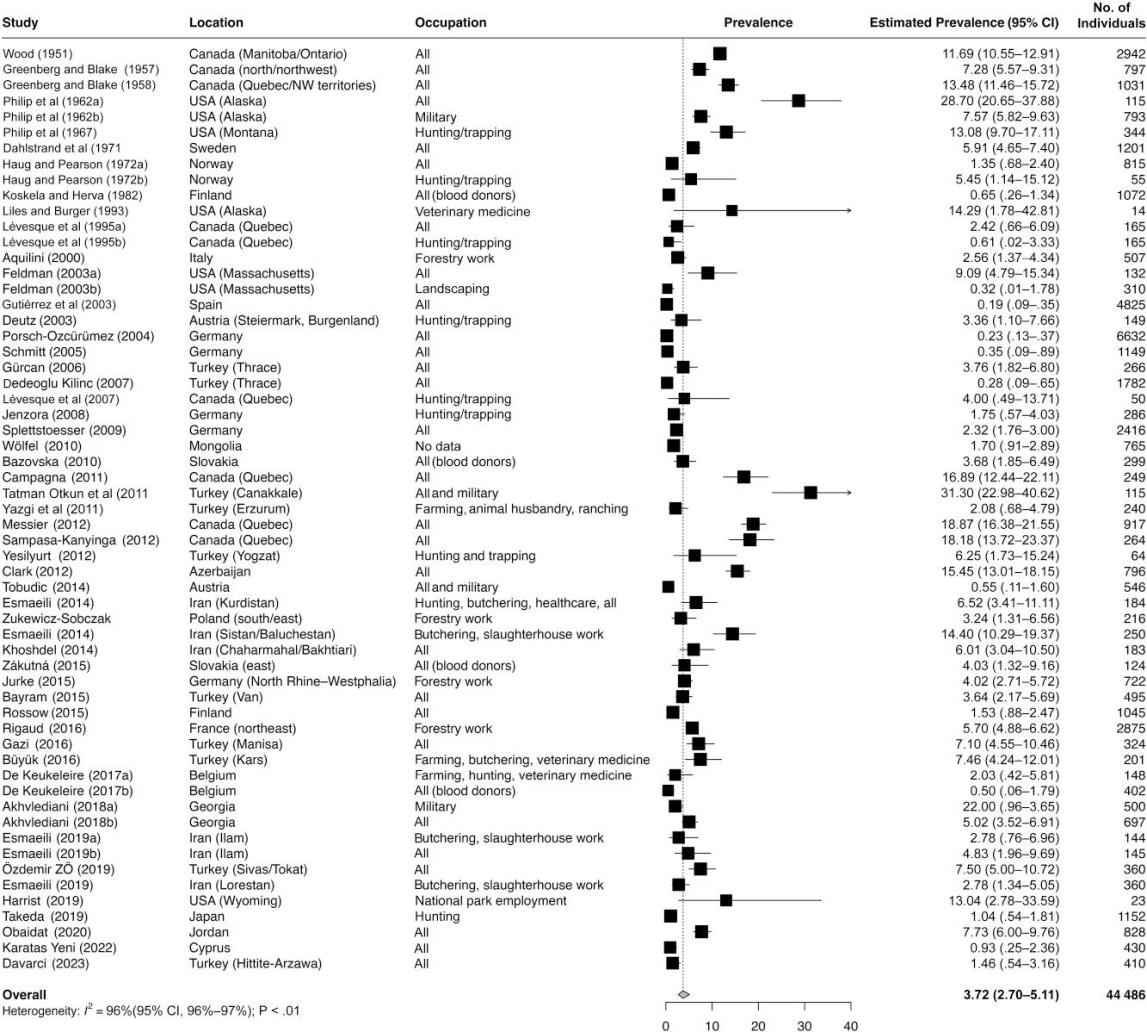
Forest plot of the pooled seroprevalence rates for antibodies against *Francisella tularensis* [6–8, 23–71]. Abbreviation: CI, confidence interval.

A total of 44 486 participants were included. Seroprevalence rates as reported in each study ranged from 0.2% to 31.3% (Table 2). Sample size) and seroprevalence rate were not significantly correlated (Pearson *r* = −0.203 [95% CI, −.436 to .056]; *P* = .12). The weighted seroprevalence rate of 59 pooled data sets calculated with the random-effects model was 3.7% (95% CI, 2.7%–5.1%) (Figure 2). The *I^2^* statistic was 96% (95% CI, 96%–97%; *P* < .01), indicating major heterogeneity. This was expected because of real between-study differences in the participant cohorts’ risk of exposure to *F tularensis*. A funnel plot designed for proportion studies [14] (Supplementary Figure 3 and Supplementary Data File 1) confirmed the heterogeneity, in that there was no convergence of prevalence rates as the sample sizes increased.

Male and female individuals accounted for 18 106 and 9885 participants, respectively, in a total of 28 data sets. Sex was not specified for another 16 477 participants in 31 data sets. The serostatus could be attributed to sex in 10 994 male and 8094 female participants (Table 3). The respective weighted seroprevalence rates were 4.7% (95% CI, 3.0%–7.3%) and 5.2% (3.1%–8.8%), and the RR was 0.83 (.66–1.05). We did not calculate age-specific pooled seroprevalence rates because the reporting of age and age group in the original studies was very heterogeneous and did not allow consistent grouping across a majority of studies.

**Table 3.** Pooled Weighted Seroprevalence of *Francisella tularensis* Antibodies According to Sex, Geographic Region, Risk of Exposure, Testing Strategy and Clinical Data Availability in Study Participants From 52 Studies Included in Review

Characteristic	Characteristic Present					Characteristic Absent							
	Participants, No.		Proportion (95% CI)			Participants, No.		Proportion (95% CI)			RR (95% CI)		
	Total	Seropositive	Raw	Fixed-Effects Model	Random-Effects Model	Total	Seropositive	Raw	Fixed-Effects Model	Random-Effects Model	Raw	Fixed-Effects Model	Random-Effects Model
Male sex^a^	10 994	566	5.1	8.8 (8.1–9.5)	4.7 (3.0–7.3)	8054	394	4.9	9.1 (8.3–10)	5.2 (3.1–8.8)	1.05 (.93–1.19)	0.99 (.92–1.05)	0.83 (.66–1.05)
North America (vs Europe or Asia)	8311	967	11.6	12.9 (12.1–13.7)	9.6 (6.3–14.3)	36175	929	2.6	5.0 (4.7–5.3)	2.7 (1.9–3.9)	4.53 (4.15–4.94)	4.53 (4.33–4.74)	4.53 (4.15–4.94)
Occupational risk of exposure	16554	1281	7.7	10.0 (9.5–10.6)	5.5 (3.9–7.8)	27932	615	2.2	5.1 (4.7–5.5)	2.4 (1.5–3.8)	3.51 (3.19–3.86)	3.51 (3.35–3.69)	3.51 (3.20–3.86)
High-specificity serologic testing used	34486	1189	3.4	7.5 (7.1–8)	3.5 (2.4–5.1)	10000	707	7.1	8.9 (8.3–9.6)	4.6 (2.6–8.1)	0.49 (.45–.54)	0.49 (.47–.51)	0.49 (.45–.53)
Clinical information available	13807	965	7.0	9.4 (8.9–10)	5.5 (3.8–7.8)	30679	931	3.0	6.8 (6.4–7.2)	2.6 (1.6–4.2)	2.30 (2.11–2.51)	2.30 (2.20–2.41)	2.30 (2.11–2.51)

Abbreviations: CI, confidence interval; RR, risk ratio.

^a^Information on sex was available only in a subset of studies.

Table 3 also lists the pooled weighted seroprevalence rates and estimated RRs according to the geographic region of the study sites, occupational risk of exposure, serologic testing strategy, and the availability of clinical information. The pooled seroprevalence of studies conducted in North America was greater than that from Europe and Asia combined (9.6% vs 2.7%; RR, 4.53 [95% CI, 4.15–4.94]). A combined total of 16 554 study participants (37%) reported an occupational risk of exposure to *F tularensis*. Occupational risk factors included hunting and trapping (16 studies), military service (5 studies), animal husbandry, farming and ranching (5 studies), butchering and slaughterhouse work (5 studies), forestry work (4) studies, veterinary medicine (3 studies), and landscaping (1 study). Included are North America participants whose lifestyle placed them at an increased risk of exposure. The pooled weighted seroprevalence of these at-risk populations was significantly greater than in studies of populations without such risk factors (5.5% vs 2.4%; RR, 3.51 [95% CI, 3.20–3.86]; Table 3). However, only 5 studies provided a complete comparative data set of individuals with versus without an increased risk of exposure and the respective seropositivity rates [41, 56, 58, 61, 65]. The pooled seropositivity rates of these studies were 2.7% (95% CI, 1.5%–4.9%) and 2.4% (1.3%–4.5%), respectively, with an RR of 1.20 (.94–1.53).

Grouping of the 52 studies according to serologic testing strategy revealed that high-specificity testing, according to our ad hoc definition (Supplementary Data File 1 and Supplementary Table 2), was associated with a lower weighted seropositivity rate than non–high-specificity testing (3.5% vs 4.6%; RR, 0.49 [.45–.53]; Table 3).

### Subclinical Tularemia

The clinical details provided for study participants testing seropositive varied widely and were predominantly limited to summary statements. A total of 28 data sets [6–8, 22–25, 29, 32, 33, 35, 40, 42–44, 48–50, 52, 55, 57, 59, 62, 63, 65–67, 72] comprised a total of 13 807 individuals for whom clinical information was available. Of these, 965 tested positive (weighted seroprevalence 5.5% [95% CI, 3.8%–7.8%] vs 2.6% [1.6%–4.2%]) in studies without clinical data) (Table 3). Supplementary Table 3 in the Supplementary Data File 1 lists pertinent quotations from the narrative clinical accounts given in each study.

**Figure 3.**
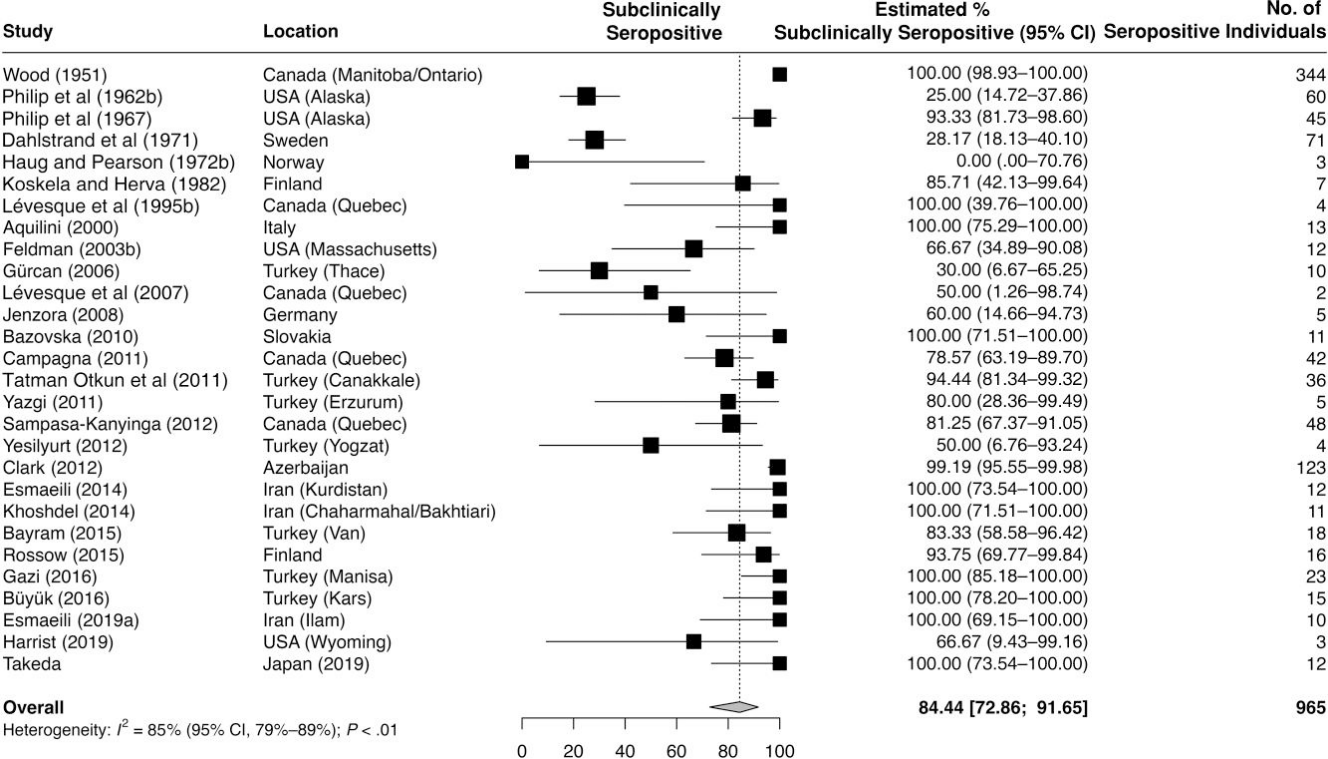
Forest plot of the pooled rates of subclinical seropositivity for antibodies against *Francisella tularensis* [6–8, 23–25, 29, 32, 33, 35, 40, 42–44, 46, 48–50, 52, 53, 55, 57, 59, 62, 63, 65–67]. Abbreviation: CI, confidence interval.

A medical history compatible with tularemia or a tularemialike illness (with or without a physician-confirmed diagnosis) among these 965 seropositive individuals could be elicited in 143, leaving 819 considered as having had subclinical tularemia. Figure 3 provides a Forest plot with the weighted rates of subclinical seropositivity reported in these studies. Overall, the weighted subclinical seropositivity rate was 84.4% (95% CI, 72.9%–91.7%). Additional subanalyses (Table 4) revealed that this rate was lower in studies from areas where, according to the literature [9, 73, 74], *F tularensis* type A was prevalent [6, 66] than in studies from type B areas. Similarly, studies from areas with a predominance of pulmonary tularemia around the time of serum sampling [6, 8] reported lower subclinical seropositivity rates than studies from areas where (ulcero)glandular or oropharyngeal tularemia prevailed. Subanalyses according to occupational risk of exposure and testing strategy are also listed in Table 4.

**Table 4.** Pooled Weighted Subclinical Seropositivity Rate for *Francisella tularensis* According to Study Location, Geographic Predominance of Type A vs Type B, Predominance of Different Clinical Syndromes in the Study Area, Presence or Absence of Occupational Risk of Exposure, and Serologic Testing Strategy

Characteristic	Characteristic Present					Characteristic Absent					RR (95% CI)		
	All seropositve	Subclincial seropositive	Proportion (95% CI)			All seropositive	Subclinical seropositive	Proportion (95% CI)					
			Raw	Fixed-Effects Model	Random -Effects Model			Raw	Fixed-Effects Model	Random-Effects Model	Raw	Fixed-Effects Model	Random-Effects Model
North America (vs Europe or Asia)	560	488	87.1	65.6 (57.6–72.9)	80.7 (54.5–93.5)	405	331	81.7	63.0 (54.5–70.8)	86.3 (72.5–93.8)	1.07 (1.01–1.13)	1.07 (1.04–1.10)	1.07 (1.01–1.13)
*Francisella tularensis* type A area (vs type B)	75	25	33.3	33.4 (23.1–45.6)	47.1 (18.7–77.6)	890	794	89.2	72.5 (66.6–77.7)	87.3 (76.7–93.5)	0.37 (.27–.51)	0.37 (.32–.44)	0.37 (.27–.51)
Predominance of pulmonary tularemia (vs [ulcero]glandular or oropharyngeal tularemia)	83	28	33.7	33.6 (23.9–44.9)	44.6 (14.2–79.8)	882	791	89.7	74.1 (68.3–79.2)	86.7 (76.1–93.1)	0.38 (.28–.51)	0.38 (.32–.44)	0.38 (.28–.51)
Occupational risk of exposure	639	559	87.5	68.3 (61.2–74.7)	82 (66.2–91.3)	326	260	79.8	57.8 (48.1–66.9)	88.1 (67.6–96.4)	1.10 (1.03–1.17)	1.10 (1.06–1.13)	1.10 (1.03–1.17)
High-specificity serologic testing	446	321	72.0	51.0 (43.7–58.2)	78.3 (61.1–89.3)	519	498	96.0	86.4 (80.3–90.8)	93.0 (82.3–97.4)	0.75 (.71–.80)	0.75 (.73–.77)	0.75 (.71–.80)

Abbreviations: CI, confidence interval; RR, risk ratio.

## DISCUSSION

This systematic review and meta-analysis covers 52 studies from North America, Europe, and Asia. Studies from North America dominated the first 50 years of observation between 1951 and 2002, while studies from Europe and Asia combined (mostly its westernmost region, including Turkey and Iran) prevailed between 2003 and 2023 (Supplementary Figures 1 and 2). The latter contributed more than three-quarters of all study subjects (Table 3), reflect the more recent regional seroepidemiology, and mainly used advanced, commercially available methods for testing. Their weighted pooled seroprevalence was >4-fold smaller than the corresponding rate derived from North American studies (Table 3). Possible explanations include differences in virulence of circulating *F tularensis* subtypes and clades, different transmission paths and dynamics, the predominance of indigenous participants in North American studies [23, 25, 29–32, 42, 43, 50, 51], whose lifestyle may have led to frequent to exposure, and possibly less specific serologic methods used in the earlier decades of the observation period. However, the absolute difference in seroprevalence being 6.9% only, we chose to pool study data in subsequent analyses irrespective of their geographic origin.

The weighted overall seroprevalence rate of 3.5% for *F tularensis* antibodies (Figure 1) and the equally important finding that 90% of reported rates ranged between 0.3% and 18% (Table 2) emphasize that only a small minority of individuals living in endemic areas provide serologic evidence of past infection. These findings were the result of pooling data that were generated by the use of different serologic assays. Our original intention to group and compare the studies according to the test type used was not feasible as the combinations of methods and cutoff values, the choices of confirmatory tests and the reporting formats varied widely (Table 2 and Supplementary Data File 2). Thus, we devised an ad hoc definition for “high-specificity” testing (Supplementary Table 2), which indeed identified a group of studies that yielded a lower pooled seroprevalence rate suggesting greater specificity than non–“high-specificity” testing (Table 3). The absolute difference of the pooled seroprevalences, grouped accordingly at 1.1% (Table 3), again was not clinically or epidemiologically relevant.

The finding of low seroprevalence rates across most of the reviewed studies is important both epidemiologically and clinically. It may reflect that tularemia transmission can be highly focal and that even within endemic areas the risk of acquisition is extremely heterogeneous. Also, as serum antibodies persist for decades, cross-sectional serosurveys capture exposure events dating back many years, underscoring the rarity of tularemia. Clinically, the interpretation of a positive serologic result in a patient is facilitated by knowing the pretest likelihood of seropositivity in the community. Even when such data are not available at a particular location, this figure indicates that the “background” seropositivity rate in endemic areas is predictably low, irrespective of the serologic test system used. A low seroprevalence was also found for study participants reporting occupational or lifestyle activities expected to increase the risk of exposure. While their likelihood of testing positive was indeed >3-fold that of not-at-risk participants (Table 3), their pooled seroprevalence rate remained low at 5.5%, with 90% of studies reporting a rate <15% (Supplementary Table 2 and Supplementary Data File 2).

The second objective of this review was to quantify the proportion of seropositive individuals who had undergone infection subclinically (ie, who did not report a history compatible with past tularemia). The use of seroprevalence studies to address the issue of subclinical infection is well established and has been used previously for other pathogens, (eg, *Borrelia burgdorferi*) [75, 76]. Our calculation indicates that this proportion was 84.4% (Figure 3) and was not affected by reported risk factors for exposure (Table 4). Interestingly, subclinical infection appears to the norm for many vector-borne zoonoses, for example, Lyme disease [75] or tick-borne encephalitis [77]. The interest in establishing the rate of subclinical tularemia is epidemiologic, clinical, and scientific. In clinical epidemiology, mandatory reporting of tularemia cases is a common public health tool used to monitor the disease activity over time. Our data suggest that this tool likely catches 10%–20% of infections at best, as subclinical cases remain unreported. Knowledge of the rate of subclinical infection thus provides a basis to roughly estimate true infection rates. Our findings also suggest, however, that such extrapolation may not be applicable in outbreak situations and when pneumonic tularemia indicates aerosol transmission [6, 8].

In clinical practice, knowing the rate of subclinical seropositivity provides additional information for the interpretation of a positive serology, even when seroconversion around the time of an acute illness appears to strongly suggest true tularemia. Considering the high rate of subclinical tularemia, the positive predictive value (PPV) of a given serologic test may be lower than commonly reported, for example, for enzyme-linked immunosorbent assays [38, 78]. Definitions for “true-positive” used to calculate the PPV often rely on compatible clinical illness (reviewed by Maurin [1]) and may lead to overestimation of the PPV, as compatible clinical illnesses, such as acute lymphadenopathy or pneumonia, have multiple causes. with tularemia being an infrequent one. Thus, when tularemia is suspected, care should be taken to identify the organism by culture or polymerase chain reaction whenever possible.

In science, an estimate of the rate of subclinical seropositivity may add a puzzle piece to what is known about host susceptibility to *F tularensis*. Our finding suggests that the immune defenses of most individuals, who mount a detectable humoral immune response, control and eliminate *F tularensis* in the absence of substantial clinical manifestations. The question arises why then a 10%–20% minority of becomes clinically ill at all. Only isolated cases in patients with defined immunodeficiencies and severe tularemia have been described (reviewed by Bahuaud and coworkers [79]). The vast majority of patients appear immunologically healthy before and after the disease. Future research could be directed toward identifying subtle deviations within the framework of virulence factors and immunoprotective events associated with tularemia that could explain a temporal susceptibility to symptomatic disease in otherwise immunocompetent individuals. This could entail a comprehensive “systems-level” approach [80], comparing immune functions between previously healthy individuals with severe tularemia and individuals with subclinical infection.

The results of this systematic review need to be taken with caution. In the former Sovjet Union, tularemia was extensively studied, but publications were inaccessible using the search strategy we used. Limitations also include the large time window of study dates and widely scattered locations, virulence differences between and within the 2 main subspecies, partly incomplete description of participant cohorts, diverse serologic test systems, and the often sketchy descriptions of how the clinical histories of seropositive participants were obtained. It also needs to be remembered that the fundamental problem of serosurveys for estimating subclinical infection rates is the often low disease prevalence in these settings. Consequently, the PPV of serology is low, with a tendency to overestimate subclinical infection because of contamination by false-positive results. In addition, as the majority of clinical information obtained was historical and reflected the participants’ recollections, it can be assumed that the data are incomplete and likely overestimate the rate of subclinical infections.

In conclusion, we find that in the temperate and arctic zones of the northern hemispheres where human tularemia occurs, only a small proportion of the population has ever been exposed to *F tularensis*; 80%–90% of exposed persons are not aware of ever having had overt tularemia or a clinical illness compatible with it.

## Supplementary Material

ofad636_Supplementary_Data

## Supplementary Data

Supplementary materials are available at *Open Forum Infectious Diseases* online. Consisting of data provided by the authors to benefit the reader, the posted materials are not copyedited and are the sole responsibility of the authors, so questions or comments should be addressed to the corresponding author.
