# Supplementary material for: Seroepidemiology of Human Tularemia—Systematic Review and Meta-analysis of Seroprevalence Studies
Source: Open Forum Infect Dis. 2023 Dec 14;11(2):ofad636. doi: 10.1093/ofid/ofad636 (PMC10837002; doi:10.1093/ofid/ofad636)
Supplement: ofad636_Supplementary_Data [file ofad636_supplementary_data.zip › OFID-D-23-01203_supplementary_data_1.docx]

**Figure S1**

Publication year and geographic origin of 52 *F. tularensis* seroprevalence studies


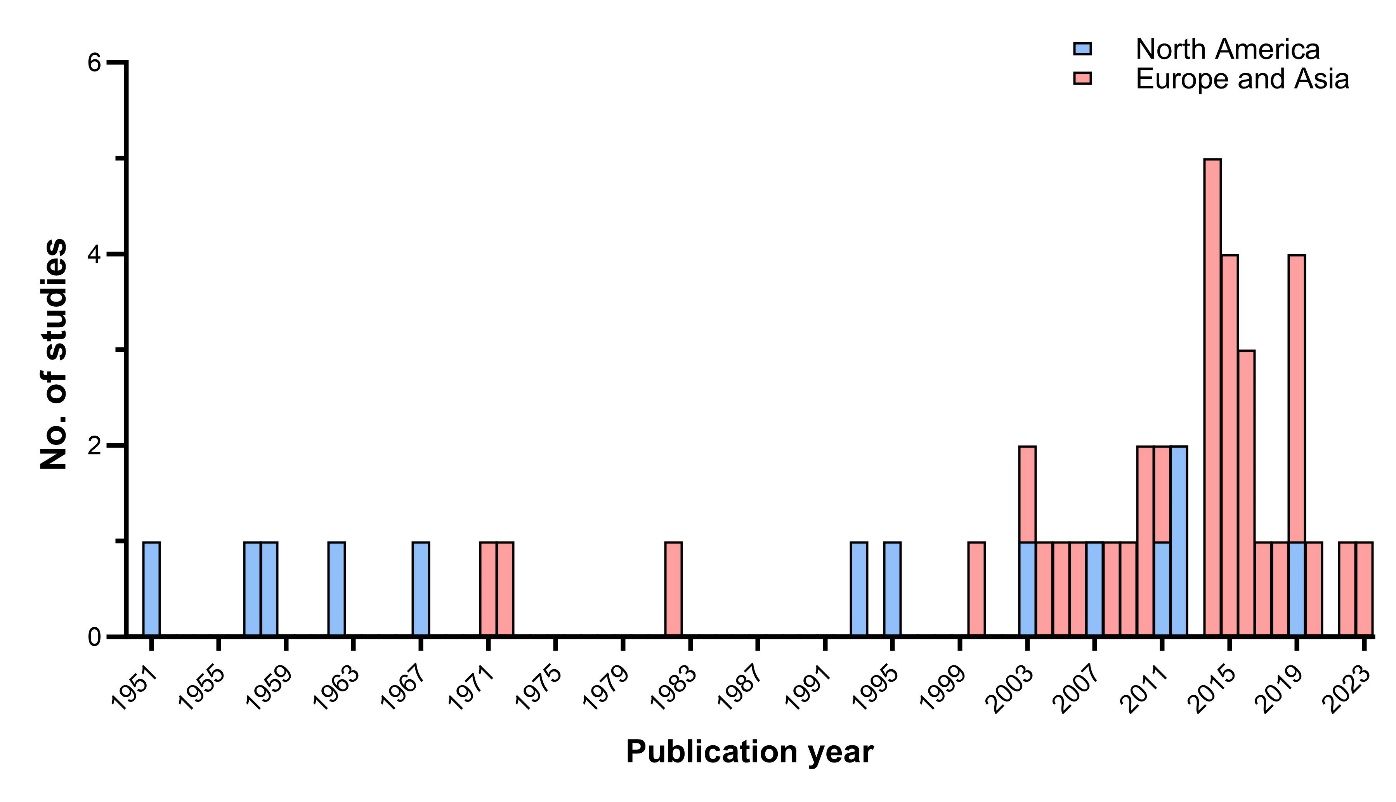


**Figure S2**

Geographic distribution of study countries of 52 reports providing human seroprevalence data for *F. tularensis*


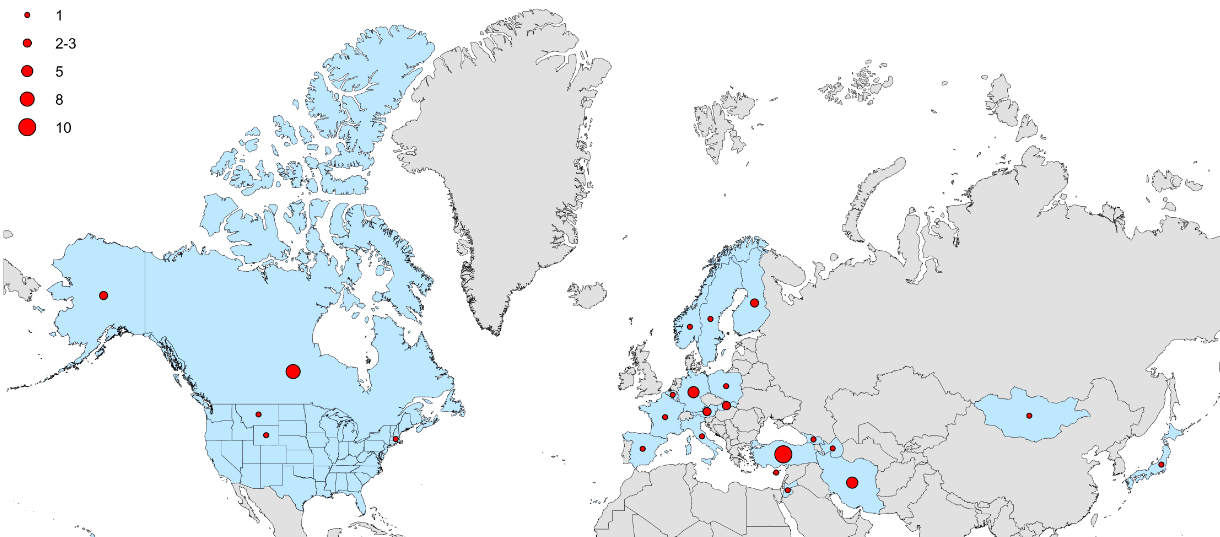


**Figure S3**

Funnel plot of *F. tularensis* seroprevalence of 59 datasets from 52 studies categorized by low vs. high serologic test specificity based on the definition detailed in table S2

**
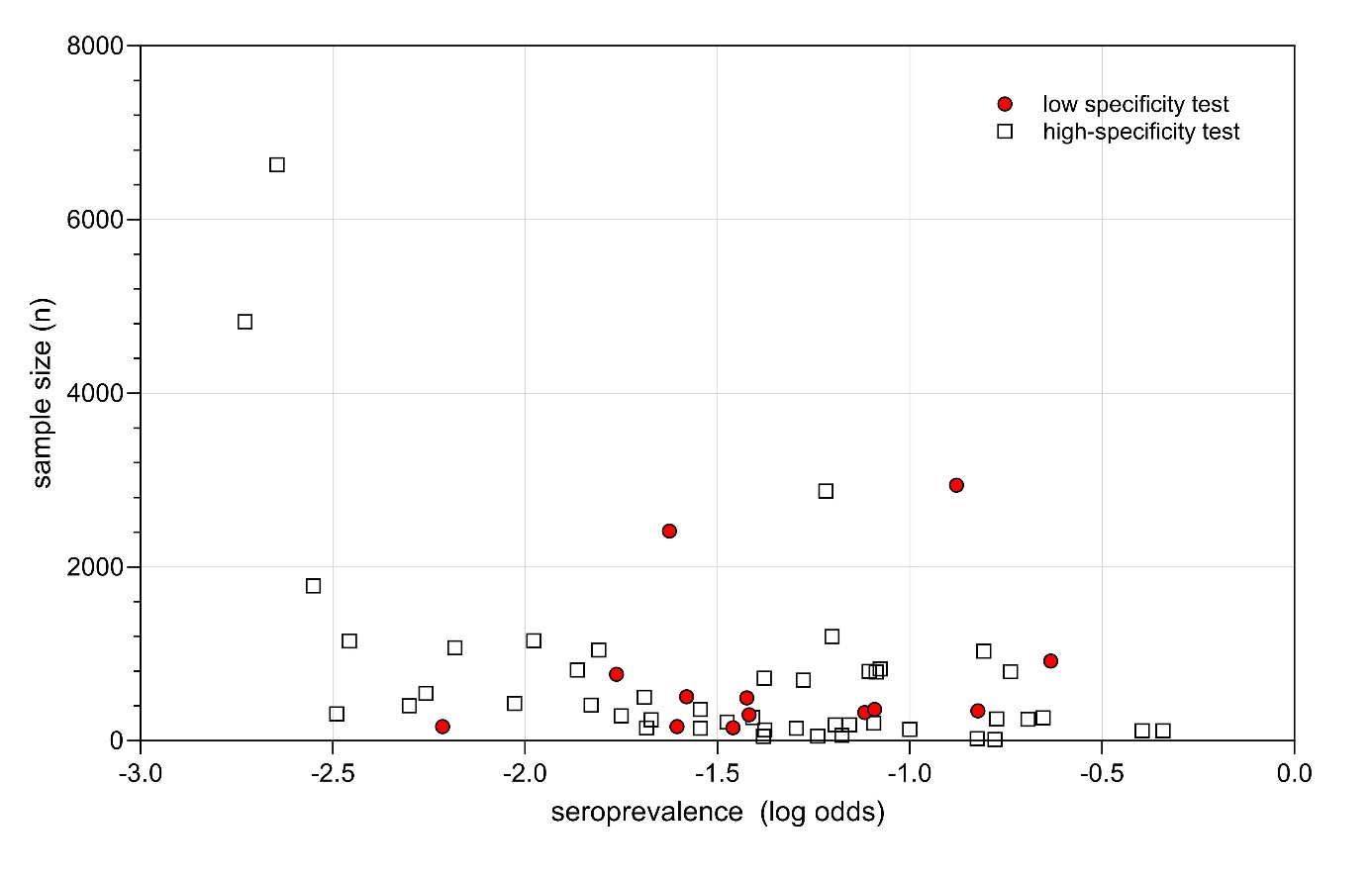
**

**Table S1**

Critical appraisal checklist adapted from the Joanna Briggs Institute risk of bias checklist [1, 2]

|  | Question | Yes | No | Unclear | Not applicable |
| --- | --- | --- | --- | --- | --- |
| 1 | Was the sample representative of the target population?  [Was the sample representative for the population whose exposure to *F. tularensis* was to be measured?] | x |  |  |  |
| 2 | Were study participants sampled in an appropriate way?  [Were individuals whose serologic test was performed excluded if done as part of a diagnostic workup for tularemia?] | x |  |  |  |
| 3 | ~~Was the sample size adequate?~~ |  |  |  |  |
| 4 | Were the study subjects and the setting described in detail?  [Sex, age, risk of exposure to *F. tularensis*, geographic region] | x |  |  |  |
| 5 | Was the data analysis conducted with sufficient coverage of the identified sample?  [Proportion of sample population that was tested?] | x |  |  |  |
| 6 | Were objective, standard criteria used for the measurement of the condition?  [Were serologic test methods described in detail?] | x |  |  |  |
| 7 | Was the condition measured reliably?  [Were all sera tested with an identical method and cut-off value?] | x |  |  |  |
| 8 | ~~Was there appropriate statistical analysis?~~ |  |  |  |  |
| 9 | ~~Are all important confounding factors, subgroups, differences identified and accounted for?~~ |  |  |  |  |
| 10 | Were subpopulations identified using objective criteria?  [Use of a questionnaire for each participant gathering clinical and epidemiologic data?] | x |  |  |  |

Questions reformulated for the purpose of this study are highlighted.

Comment: Item 3 was omitted because no minimal sample size was needed for study inclusion. Item 8 was omitted because the calculation of seroprevalence rates did not require statistical expertise and could be recalculated for the purpose of this project. Item 9 was omitted because inclusion criteria did not require subgrouping according to specified criteria.

**Table S2**

Definition of high-quality serologic test strategies for the purpose of this study

For the purpose of this systematic review we defined that high-quality a test strategy required at least 1 of the following criteria:

- TAT ≥80^1^
- TAT ≥20^1^ plus cross-reactivity test for *Brucella sp.* antibodies
- MAT ≥128^1^
- MAT ≥20^1^ plus cross-reactivity test for *Brucella sp.* antibodies
- any test confirmed by WB
- any commercial ELISA (Serion® or Seramun®) according to the manufacturer’s instruction and cut-off recommendation

^1^ reciprocal of serum dilution

Abbreviations

TAT: tube agglutination test; MAT: microagglutionation test; WB: Westerblot/Immunoblot; ELISA: enzyme-linked immunosorbent assay

**Table S3**

Narrative excerpts of clinical information provided on study participants seropositive for *F. tularensis*

| **Study** | **Quotation** |
| --- | --- |
| Wood WJ (1951) [3] | "An unusual aspect of this survey … was the absence of any reported cases of clinical disease."  “Personal inquiry of Indians having very high titers revealed no history of disease …”  “No cases of clinical tularemia in Indians were seen." |
| Greenberg L (1957) [4] | None, |
| Greenberg L (1957) [5] | None. |
| Philip RN (1962) [6] | "The table is noteworthy for the paucity of information concerning illnesses resembling clinical tularemia. There were no differences either in total illnesses or tularemia-like illnesses in the groups with and without antibodies. Even 2 persons with agglutinin titers of 640 did not have illnesses suggestive of tularemia. The majority of illnesses were mild, and only 5 were sufficiently important to require medical attention. One of the 5 illnesses was subsequently diagnosed as pulmonary tuberculosis; the other 4 were undiagnosed. Two of these patients had "sores on the hands" after skinning muskrats; another was treated for "an infected mosquito bite on the left foot"; and one was hospitalized for 10 days because of a "boil on the right leg." Perhaps several of these illnesses were tularemia, but the evidence is not convincing." |
| Philip RN (1967) [7] | "Twenty-two per cent of reactors and only 2% of nonreactors had had confirmed tularemia or tularemia-like illnesses. Of (…these…) 13 reactors with such illnesses 3 had had serologically confirmed tularemia.”  “Many of these were clinically ill defined or were not characteristic of tularemia. However, if the clinical information is reasonably accurate, then roughly three-quarters of the infections in the trapper group were inapparent or atypical." |
| Dahlstrand S (1971) [8] | Detailed description of clinical manifestations in 402 cases provided in Tables VII-IX.  Table VI provides an estimate of the proportion of subclinical cases in a subset of 84 patients (20 cases were subclinical). |
| Haug RH (1972) [9] | "As concerns the occurrence of overt clinical tularemia in relation to the serological results, the study seems to indicate a difference between the school group and the hunters' group. In the latter group, all three individuals showing positive agglutination are known to have had tularemia. In the school groups no information of clinically diagnosed tularemia has been obtained during the collective examination in the schools." |
| Koskela P (1982) [10] | "Of the 5 persons with definite immunity to *F. tularensis*, 4 had been without symptoms serious enough to be remembered years or decades later." |
| Liles WC (1993) [11] | None. |
| Levesque B (1995) [12] | "The medical histories documented by the questionnaire were negative, except for three trappers who reported a prior tularemia episode but were seronegative in the study." |
| Aquilini D (2000) [13] | "None of the subjects examined reported having had previous specific clinical symptoms compatible with the diseases studied in this work." |
| Feldman KA (2003) [14] | "Two seropositive landscapers reported having been diagnosed with tularemia by a physician …; two others reported having had an undiagnosed febrile illness in 2000 or 2001."  "Eight of the seropositive landscapers did not report a previous diagnosis of tularemia or recall an undiagnosed febrile illness in 2000 or 2001." |
| Gutierrez MP (2003) [15] | None. |
| Deutz A (2003) [16] | None. |
| Porsch-Ozcürümez M (2004) [17] | None. |
| Schmitt P (2005) [18] | None. |
| Gürcan S (2006) [19] | "Seven villagers had an oropharyngeal form of tularemia. Four and three patients had the antibody against F. tularensis at 1:5,120 and 1:10,240 dilutions, respectively. Also, three asymptomatic cases were detected with 1:160 -1:5,120 antibody dilutions. Four of the 10 cases belonged to the same family; the other 6 were each from a different family." |
| Dedeoglu Kilinc G (2007) [20] | None. |
| Levesque B (2007) [21] | "One subject … with positive *F. tularensis* serology had been treated for an infected inguinal lymph node." |
| Jenzora A (2008) [22] | "Two hunters reported clinical symptoms: one hunter experienced severe pneumonia 2 years ago. At that time, tularemia was not considered as diagnosis. The second hunter suffered from chronic muscle pain and hepatitis of unknown origin. No clinical symptoms were reported from the remaining three hunters." |
| Splettstoesser WD (2009) [23] | None. |
| Bazovska S (2010) [24] | "These were persons who had not been reported to have the disease in the context of the surveillance of these infections."^1^ |
| Wölfel R (2010) [25] | None. |
| Campagna S (2011) [26] | "A review of medical records revealed very few participants with a documented history of symptoms compatible with the particular infections suggested by serologic results."  "Nine participants (9/42; 21.4%) seropositive for *F. tularensis* had sought medical attention for symptoms that could have been associated with clinical tularemia: 3 were diagnosed with conjunctival infections and 6 with pharyngeal infections including one with cervical adenopathy who tested negative for mononucleosis" |
| Otkun M (2011) [27] | "The two cases numbered 9 and 22 … , whose characteristics are given in Table I, lived together at home."  "Case 9: 9 year old boy with left submandibular lymph node. Case 22: 25 years old housewife with bilateral multiple lymph nodes." ^1^ |
| Yazgi H (2011) [28] | "Two of the three volunteers did not give a history of a clinical picture suggestive of tularemia in the past, and one described a disease two years ago with clinical findings resembling the oropharyngeal form." ^1^ |
| Sampasa-Kanyinga H (2012) [29] | "1 case with atypical pneumonia, 4 cases pharyngitis, 1 case with pneumonia, 3 cases with conjunctivitis (Table 5).” |
| Messier V (2012) [30] | None. |
| Yesilyurt M (2012) [31] | "When the seropositive cases were evaluated clinically, two hunters with antibody titer 1/160 did not have any complaints compatible with tularemia infection (febrile illness, sore throat and/or conjunctivitis, lymph node enlargement-pain or skin ulcers/wounds, etc.) in their history, while one case described clinical signs and symptoms compatible with oropharyngeal form two years ago. The hunter with an antibody titer of 1/2560 developed acute oropharyngeal tularemia." ^1^ |
| Clark DV (2012) [32] | "Few subjects reported a history of lymphadenopathy in this population (8), only 1 of which was serologically positive for tularemia. No clinical symptoms were associated with tularemia seropositivity." |
| Tobudic S (2014) [33] | None. |
| Esmaeili S (2014) (Iran) [34] | None. |
| Zukiewicz-Sobczak W (2014) [35] | None. |
| Esmaeili S (2014) [36] | "Because the seropositive participants were asymptomatic, positive antibody titers probably indicated past tularemia infection." |
| Khoshdel A (2014) [37] | "Volunteers had not fever, skin sore, lymphadenopathy or other sign and symptom of disease." |
| Zákutná L (2015) [38] | None. |
| Jurke A (2015) [39] | None. |
| Bayram Y (2015) [40] | "Detailed questioning of these 14 individuals revealed that 3 of them had been diagnosed and treated for tularemia previously."  "Three individuals stated that they had been treated for oropharyngeal tularemia in the past. No evidence of the disease was found in other individuals." ^1^ |
| Rossow H (2015) [41] | "One of them reported being hospitalised because of tularemia during the period from 1995 to 2000 (precise time and duration of hospitalisation not available). |
| Rigaud E (2016) [42] | None. |
| Gazi H (2016) [43] | "All the subjects included in the study were healthy and had no signs of infection." |
| Büyük F (2016) [44] | "No clinical complaints related to tularemia had been reported previously by the volunteers" |
| De Keukeleire M (2017) [45] | None. |
| Akhvlediani N (2018) [46] | None. |
| Esmaeili S (2019) [47] | None. |
| Özdemir ZÖ (2019) [48] | None. |
| Esmaeili S (2019) [49] | "No present or past history of tularemia could be recorded in seropositive individuals."  "Referents of medical diagnostic laboratories (healthy people without any clinical symptoms) from the same area were selected as the control group." |
| Harrist A (2019) [50] | "One participating employee was the patient diagnosed with tularemia in August."  "Neither of the two seropositive employees identified in the serosurvey reported ever being diagnosed with tularemia, being diagnosed with pneumonia or having a festering sore in the 3 years before, or having a febrile illness during the past 3 months." |
| Takeda T (2019) [51] | "At the time of sampling, each participant was in healthy condition with no subjective symptoms." |
| Obaidat MM (2020) [52] | None. |
| Karatas Yeni D (2022) [53] | None. |
| Davarci I (2023) [54] | None. |

^1^ translated from original language to English using Deepl® pro software.

**References**

1. Munn Z, Moola S, Lisy K, Riitano D, Tufanaru C. Methodological guidance for systematic reviews of observational epidemiological studies reporting prevalence and cumulative incidence data. Int J Evid Based Healthc **2015**; 13(3): 147-53.

2. Munn Z, Moola S, Riitano D, Lisy K. The development of a critical appraisal tool for use in systematic reviews addressing questions of prevalence. Int J Health Policy Manag **2014**; 3(3): 123-8.

3. Wood WJ. Tularemia; a study based on the incidence of positive agglutination tests against P. tularensis in the Indian population of Manitoba and North-Western Ontario. Manit Med Rev **1951**; 31(10): 641-4.

4. Greenberg L, Blake JD. An immunological study of the Canadian Indian. Can Med Assoc J **1957**; 77(3): 211-6.

5. Greenberg L, Blake JD. An immunological study of the Canadian Eskimo. Can Med Assoc J **1958**; 78(1): 27-31.

6. Philip RN, Huntley B, Lackman DB, Comstock GW. Serologic and skin test evidence of tularemia infection among Alaskan Eskimos, Indians and Aleuts. J Infect Dis **1962**; 110: 220-30.

7. Philip RN, Casper EA, Lackman DB. The skin test in an epidemiologic study of tularemia in Montana trappers. J Infect Dis **1967**; 117(5): 393-402.

8. Dahlstrand S, Ringertz O, Zetterberg B. Airborne tularemia in Sweden. Scand J Infect Dis **1971**; 3(1): 7-16.

9. Haug RH, Pearson AD. Human infections with Francisella tularensis in Norway. Development of a serological screening test. Acta Pathol Microbiol Scand B Microbiol Immunol **1972**; 80(2): 273-80.

10. Koskela P, Herva E. Immunity against Francisella tularensis in northern Finland. Scand J Infect Dis **1982**; 14(3): 195-9.

11. Liles WC, Burger RJ. Tularemia from domestic cats. West J Med **1993**; 158(6): 619-22.

12. Levesque B, De Serres G, Higgins R, et al. Seroepidemiologic study of three zoonoses (leptospirosis, Q fever, and tularemia) among trappers in Quebec, Canada. Clin Diagn Lab Immunol **1995**; 2(4): 496-8.

13. Aquilini D, Parola, P. Salvo, E., Paladini, A. Seroepidemiology of the rickettsioses, human granulocytic ehrlichiosis, Lyme disease, Q fever, and tularemia in forestry workers in Tuscany, Italy. Journal of Spirochetal and Tick-borne Diseases **2000**; 7(Fall): 35-41.

14. Feldman KA, Stiles-Enos D, Julian K, et al. Tularemia on Martha's Vineyard: seroprevalence and occupational risk. Emerg Infect Dis **2003**; 9(3): 350-4.

15. Gutierrez MP, Bratos MA, Garrote JI, et al. Serologic evidence of human infection by Francisella tularensis in the population of Castilla y Leon (Spain) prior to 1997. FEMS Immunol Med Microbiol **2003**; 35(2): 165-9.

16. Deutz A, Fuchs K, Nowotny N, et al. [Sero-epidemiological studies of zoonotic infections in hunters--comparative analysis with veterinarians, farmers, and abattoir workers]. Wien Klin Wochenschr **2003**; 115 Suppl 3: 61-7.

17. Porsch-Ozcurumez M, Kischel N, Priebe H, Splettstosser W, Finke EJ, Grunow R. Comparison of enzyme-linked immunosorbent assay, Western blotting, microagglutination, indirect immunofluorescence assay, and flow cytometry for serological diagnosis of tularemia. Clin Diagn Lab Immunol **2004**; 11(6): 1008-15.

18. Schmitt P, Splettstosser W, Porsch-Ozcurumez M, Finke EJ, Grunow R. A novel screening ELISA and a confirmatory Western blot useful for diagnosis and epidemiological studies of tularemia. Epidemiol Infect **2005**; 133(4): 759-66.

19. Gurcan S, Eskiocak M, Varol G, et al. Tularemia re-emerging in European part of Turkey after 60 years. Jpn J Infect Dis **2006**; 59(6): 391-3.

20. Dedeoglu Kilinc G, Gurcan S, Eskiocak M, Kilic H, Kunduracilar H. [Investigation of tularemia seroprevalence in the rural area of Thrace region in Turkey]. Mikrobiyol Bul **2007**; 41(3): 411-8.

21. Levesque B, Messier V, Bonnier-Viger Y, et al. Seroprevalence of zoonoses in a Cree community (Canada). Diagn Microbiol Infect Dis **2007**; 59(3): 283-6.

22. Jenzora A, Jansen A, Ranisch H, Lierz M, Wichmann O, Grunow R. Seroprevalence study of Francisella tularensis among hunters in Germany. FEMS Immunol Med Microbiol **2008**; 53(2): 183-9.

23. Splettstoesser WD, Piechotowski I, Buckendahl A, et al. Tularemia in Germany: the tip of the iceberg? Epidemiol Infect **2009**; 137(5): 736-43.

24. Bazovska S, Vyrostekova, G.D., Jarekova, J., Bakoss, P., Machacova, E., Spalekova, M. Antibodies against the causative agents of some natural focal infections in blood donor sera from western Slovakia. Epidemiol Mikrobiol Imunol **2010**; 59(4): 168-71.

25. Wölfel R, Altantuul, D., Mossbrugger, I., Zorig, L., Enkhtuvshin, B., Davaadorj, R. . Seroprevalence of zoonoses in Mongolia: Surveillance and risk factor assessment. American Journal of Tropical Medicine and Hygiene **2010**; 83: 146.

26. Campagna S, Levesque B, Anassour-Laouan-Sidi E, et al. Seroprevalence of 10 zoonotic infections in 2 Canadian Cree communities. Diagn Microbiol Infect Dis **2011**; 70(2): 191-9.

27. Tatman Otkun M, Akcali A, Karadenizli A, et al. [Epidemiological evaluation of a rapidly-prevented tularemia outbreak in Canakkale province, Turkey]. Mikrobiyol Bul **2011**; 45(1): 48-57.

28. Yazgi H, Uyanik MH, Ertek M, et al. [Tularemia seroprevalence in the risky population living in both rural and urban areas of Erzurum]. Mikrobiyol Bul **2011**; 45(1): 67-74.

29. Sampasa-Kanyinga H, Levesque B, Anassour-Laouan-Sidi E, et al. Zoonotic infections in native communities of James Bay, Canada. Vector Borne Zoonotic Dis **2012**; 12(6): 473-81.

30. Messier V, Levesque, B., Proulx, J.F., Rochette, L., Serhir, B., Couillard, M., Ward, B.J., Libman, M.D., Dewailly, E., Dery, S. Seroprevalence of seven zoonotic infections in Nunavik, Quebec (Canada). Zoonoses Public Health **2012**; 59(107-117): 107.

31. Yesilyurt M, Kilic S, Celebi B, Gul S. [Tularemia: are hunters really a risk group?]. Mikrobiyol Bul **2012**; 46(1): 153-5.

32. Clark DV, Ismailov A, Seyidova E, et al. Seroprevalence of tularemia in rural Azerbaijan. Vector Borne Zoonotic Dis **2012**; 12(7): 558-63.

33. Tobudic S, Nedomansky K, Poeppl W, et al. Seroprevalence for Coxiella burnetii, Francisella tularensis, Brucella abortus and Brucella melitensis in Austrian adults: a cross-sectional survey among military personnel and civilians. Ticks Tick Borne Dis **2014**; 5(3): 315-7.

34. Esmaeili S, Gooya MM, Shirzadi MR, et al. Seroepidemiological survey of tularemia among different groups in western Iran. Int J Infect Dis **2014**; 18: 27-31.

35. Zukiewicz-Sobczak W, Zwolinski J, Chmielewska-Badora J, et al. Prevalence of antibodies against selected zoonotic agents in forestry workers from eastern and southern Poland. Ann Agric Environ Med **2014**; 21(4): 767-70.

36. Esmaeili S, Esfandiari B, Maurin M, et al. Serological survey of tularemia among butchers and slaughterhouse workers in Iran. Trans R Soc Trop Med Hyg **2014**; 108(8): 516-8.

37. Khoshdel A, Saedi Dezaki, E., Ganji, F., Habibian, R., Imani, R., Taheri, E., Nikkhah, A. First seroprevalence survey of children with tularemia infection in Chaharmahal va Bakhtiari Province, Iran. Iranian Journal of Pathology **2014**; 9(1): 23-7.

38. Zakutna L, Dorko E, Rimarova K, Kizekova M. Pilot Cross-Sectional Study of Three Zoonoses (Lyme Disease, Tularaemia, Leptospirosis) among Healthy Blood Donors in Eastern Slovakia. Cent Eur J Public Health **2015**; 23(2): 100-6.

39. Jurke A, Bannert N, Brehm K, et al. Serological survey of Bartonella spp., Borrelia burgdorferi, Brucella spp., Coxiella burnetii, Francisella tularensis, Leptospira spp., Echinococcus, Hanta-, TBE- and XMR-virus infection in employees of two forestry enterprises in North Rhine-Westphalia, Germany, 2011-2013. Int J Med Microbiol **2015**; 305(7): 652-62.

40. Bayram Y, Ozkacmaz A, Parlak M, Basbugan Y, Kilic S, Guducuoglu H. [Seroprevalence of tularemia in risk groups of humans and animals in Van, eastern Turkey]. Mikrobiyol Bul **2015**; 49(4): 532-41.

41. Rossow H, Ollgren J, Hytonen J, et al. Incidence and seroprevalence of tularaemia in Finland, 1995 to 2013: regional epidemics with cyclic pattern. Euro Surveill **2015**; 20(33): 21209.

42. Rigaud E, Jaulhac B, Garcia-Bonnet N, et al. Seroprevalence of seven pathogens transmitted by the Ixodes ricinus tick in forestry workers in France. Clin Microbiol Infect **2016**; 22(8): 735 e1-9.

43. Gazi H, Ozkutuk N, Ecemis O, et al. Seroprevalence of West Nile virus, Crimean-Congo hemorrhagic fever virus, Francisella tularensis and Borrelia burgdorferi in rural population of Manisa, western Turkey. J Vector Borne Dis **2016**; 53(2): 112-7.

44. Buyuk F, Celebi O, Celik E, et al. The prevalence of tularemia in occupational groups that have contact with animals. Turk J Med Sci **2016**; 46(2): 451-6.

45. De Keukeleire M, Vanwambeke SO, Cochez C, et al. Seroprevalence of Borrelia burgdorferi, Anaplasma phagocytophilum, and Francisella tularensis Infections in Belgium: Results of Three Population-Based Samples. Vector Borne Zoonotic Dis **2017**; 17(2): 108-15.

46. Akhvlediani N, Burjanadze I, Baliashvili D, et al. Tularemia transmission to humans: a multifaceted surveillance approach. Epidemiol Infect **2018**; 146(16): 2139-45.

47. Esmaeili S, Bagheri Amiri F, Mokhayeri H, et al. Seroepidemiological study of Q fever, brucellosis and tularemia in butchers and slaughterhouses workers in Lorestan, western of Iran. Comp Immunol Microbiol Infect Dis **2019**; 66: 101322.

48. Özdemir ZÖ, Günes, T., Oyardi, Ö. Risk factors associated with the frequency of antibodies to Francisella tularensis in two areas from Turkey. Istanbul Journal of Pharmacology **2019**; 49(3): 137-41.

49. Esmaeili S, Ghasemi A, Naserifar R, et al. Epidemiological survey of tularemia in Ilam Province, west of Iran. BMC Infect Dis **2019**; 19(1): 502.

50. Harrist A, Cherry C, Kwit N, et al. Francisella tularensis Exposure Among National Park Service Employees During an Epizootic: Devils Tower National Monument, Wyoming, 2015. Vector Borne Zoonotic Dis **2019**; 19(5): 316-22.

51. Takeda T, Fujita H, Iwasaki M, et al. Positive rates of anti-acari-borne disease antibodies of rural inhabitants in Japan. J Vet Med Sci **2019**; 81(5): 758-63.

52. Obaidat MM, Malania L, Bani Salman AE, Arner RJ, Roess AA. Seroepidemiology, Spatial Distribution, and Risk Factors of Francisella tularensis in Jordan. Am J Trop Med Hyg **2020**; 103(2): 659-64.

53. Karatas Yeni D, Ruh, E., Bostanci, A., Celebi, B., Taylan Ozkan, A. Investigation of seropositivity of tularemia, brucellosis and leptospirosis in humans in northern Cyprus. Fresenius Environmental Bulletin **2022**; 31 (02/2022): 2153-60.

54. Davarci I, Eryildiz C, Renders DP, Berberoglu U, Gurcan S. Tularemia seroprevalence in humans in the region of the Hittite-Arzawa War (Inner Aegean Region), where the first biological weapon was used 3300 years ago. Turk J Med Sci **2023**; 53(1): 310-5.
